# Automated hippocampal segmentation in patients with epilepsy: Available free online

**DOI:** 10.1111/epi.12408

**Published:** 2013-10-23

**Authors:** Gavin P Winston, M Jorge Cardoso, Elaine J Williams, Jane L Burdett, Philippa A Bartlett, Miklos Espak, Charles Behr, John S Duncan, Sebastien Ourselin

**Affiliations:** * Epilepsy Society MRI Unit, Department of Clinical and Experimental Epilepsy, UCL Institute of NeurologyLondon, United Kingdom; † Centre for Medical Imaging Computing, UCLLondon, United Kingdom; Dementia Research Centre, Department of Neurodegenerative Disease, UCL Institute of NeurologyLondon, United Kingdom

**Keywords:** Hippocampal segmentation, Hippocampal sclerosis, Epilepsy surgery, Magnetic resonance imaging

## Abstract

**Purpose:**

Hippocampal sclerosis, a common cause of refractory focal epilepsy, requires hippocampal volumetry for accurate diagnosis and surgical planning. Manual segmentation is time-consuming and subject to interrater/intrarater variability. Automated algorithms perform poorly in patients with temporal lobe epilepsy. We validate and make freely available online a novel automated method.

**Methods:**

Manual hippocampal segmentation was performed on 876, 3T MRI scans and 202, 1.5T scans. A template database of 400 high-quality manual segmentations was used to perform automated segmentation of all scans with a multi-atlas–based segmentation propagation method adapted to perform label fusion based on local similarity to ensure accurate segmentation regardless of pathology. Agreement between manual and automated segmentations was assessed by degree of overlap (Dice coefficient) and comparison of hippocampal volumes.

**Key Findings:**

The automated segmentation algorithm provided robust delineation of the hippocampi on 3T scans with no more variability than that seen between different human raters (Dice coefficients: interrater 0.832, manual vs. automated 0.847). In addition, the algorithm provided excellent results with the 1.5T scans (Dice coefficient 0.827), and automated segmentation remained accurate even in small sclerotic hippocampi. There was a strong correlation between manual and automated hippocampal volumes (Pearson correlation coefficient 0.929 on the left and 0.941 on the right in 3T scans).

**Significance:**

We demonstrate reliable identification of hippocampal atrophy in patients with hippocampal sclerosis, which is crucial for clinical management of epilepsy, particularly if surgical treatment is being contemplated. We provide a free online Web-based service to enable hippocampal volumetry to be available globally, with consequent greatly improved evaluation of those with epilepsy.

The hippocampus is located within the medial temporal lobe and plays a key role in learning and episodic, semantic, and spatial memory. Dysfunction has been reported in neurologic and psychiatric disorders including epilepsy (Wu et al., 2005), Alzheimer's disease (Apostolova et al., 2006), schizophrenia (Tanskanen et al., 2005), and depression (Bremner et al., 2000).

Temporal lobe epilepsy (TLE) is the most common drug-resistant focal epilepsy, with seizures frequently arising from the hippocampus. In surgical series of TLE, the pathology is often hippocampal sclerosis (HS) comprising neuronal loss and gliosis and marked by atrophy and signal change on magnetic resonance imaging (Van Paesschen, 2004). Atrophy of the hippocampus through HS provides a good biomarker for the laterality of the seizure focus (Bernasconi et al., 2003), and combined with concordant neurophysiology and neuropsychological data can be sufficient to recommend surgery. Hippocampal atrophy is associated with a favorable surgical outcome (Schramm & Clusmann, 2008).

Visual assessment of hippocampal volumes is unreliable, as it may be compromised by head position and primarily detects hippocampal asymmetry rather than volume loss, making bilateral atrophy difficult to identify. Hippocampal segmentation and volumetry are thus important for diagnosis and surgical planning (Watson et al., 1997). The gold standard for hippocampal segmentation is manual delineation by trained raters. This is accurate, reproducible, and sensitive but is time-consuming, requires anatomic knowledge, and is subject to interrater and intrarater variability. The hippocampus is challenging to delineate as it is small and highly variable with ill-defined margins. Many protocols exist for manual segmentation depending on which structures are included and the boundary definition (Konrad et al., 2009).

Automated segmentation techniques aim to ensure operator independence, high reproducibility, and reduced demand for human time and expertise. The strongest drive for automation has come from researchers working with large cohorts of patients with Alzheimer's disease patients. Hippocampal volumes are an early marker for the disease, are related to cognitive status, and may reflect disease progression in clinical trials (Frisoni & Jack, 2011).

In atlas-based segmentation approaches, a template and associated manual labels are registered (matched) to the new image (Carmichael et al., 2005). Commonly used methods, including FreeSurfer (Fischl et al., 2002), rely on a single template so that subjects that differ significantly from the template, for example HS, are poorly segmented. Segmentation of hippocampi that are sclerotic is more challenging than segmenting hippocampi in Alzheimer's disease, as the latter is associated with more prominent cerebrospinal fluid (CSF)–hippocampal boundaries, whereas the former is associated with signal change.

The use of an atlas with multiple template images is more effective than a single template (Heckemann et al., 2006) and depends on the quality of registration and template selection strategy. Most previous atlas-based segmentation studies used small template databases of healthy subjects. Results obtained in TLE are significantly worse than in healthy subjects or Alzheimer's disease (Kim et al., 2012), as aside from atrophy, approximately 40% of patients with TLE demonstrate an atypical shape or position of the hippocampus (Bernasconi et al., 2005).

In this study, we adapted our published method developed for use in Alzheimer's disease (Cardoso et al., 2013) to a large cohort of adult patients with epilepsy by employing accurate nonlinear registration (Modat et al., 2010) and a large template database that encompasses the range of pathology observed in epilepsy at a tertiary referral center. Manual segmentations of the most similar images from the template database are combined using a label fusion strategy based on local similarity to ensure accurate segmentation regardless of pathology. We demonstrate that this technique achieves reliable segmentation with no more variability than that seen between different expert raters. The algorithm is made freely available via an online Web-based service (https://hipposeg.cs.ucl.ac.uk). In addition, the software, scripts, and an anonymized version of the template database are available from this website.

## METHODS

### Subjects

The Epilepsy Society MRI Unit opened in 1995 with a 1.5T General Electric Horizon EchoSpeed scanner (General Electric, Waukesha, WI, U.S.A.), and upgraded to a 3T General Electric Signa Excite HDx scanner (General Electric) in 2004. A dedicated epilepsy protocol magnetic resonance imaging (MRI) acquisition was used throughout.

In clinical practice, manual hippocampal volumetry is performed according to a standardized protocol. All clinical scans acquired on the 3T scanner between July 2004 and April 2012 with bilateral manual segmentations performed with this protocol were retrieved (n = 884). Three scans were excluded due to a nonstandard acquisition, and five scans were excluded due to temporal lobe surgery involving the hippocampus.

To validate the method using scans from a different scanner and field strength, all clinical scans acquired on the 1.5T scanner between May 2001 (when the manual segmentation protocol was introduced) and June 2004 with bilateral manual hippocampal segmentations were retrieved (n = 205). Three scans were excluded due to surgery involving the hippocampus.

Fifty healthy controls underwent scans on the 3T scanner, and a subset of these (18 subjects) was segmented twice by each of the two raters over a period of 3 months to assess interrater and intrarater reliability of manual segmentation.

This study was approved by the local research ethics committee. Because the study involved processing of anonymized data that had been acquired previously, individual patient consent was not required. All healthy controls provided written informed consent.

### Image acquisition

MRI studies performed on the 3T GE scanner used standard imaging gradients (maximum strength of 40 mT/m, slew rate 150 T/m/s), a body-coil for transmission, and an 8-channel phased-array coil for reception. Standard clinical sequences included a coronal T_1_-weighted volumetric fast spoiled gradient echo (SPGR) acquisition (170 contiguous 1.1 mm thick slices, matrix 256 × 256, field-of-view 24 cm, in-plane resolution 0.9375 × 0.9375 mm, acquisition time 7 min 30 s). Six scans were acquired with thicker 1.5 mm slices and six were acquired with in-plane resolution 1.0938 × 1.0938 mm. These were included in the analysis, as they represent standard clinical practice but were not used for the template database.

On the 1.5T GE scanner, data were acquired with standard imaging gradients (maximum strength of 22 mT/m and slew rate 120 T/m/s) and a circularly-polarized birdcage head-coil for transmission and reception. Clinical sequences included a T_1_-weighted volumetric inversion recovery-prepared spoiled gradient echo (IR-SPGR) sequence (124 contiguous 1·5 mm thick slices, matrix 256 × 192, field-of-view 24 × 18 cm, in-plane resolution 0.9375 × 0.9375 mm, acquisition time 6 min 56 s).

### Manual segmentation

Manual segmentations were performed using MReg, an in-house software package (Lemieux et al., 2000). Each hippocampus was manually outlined on alternate coronal oblique slices (3T) or every slice (1.5T) viewed at 4× magnification by an experienced operator (EW/JB). The entire length of each hippocampus was segmented according to previously described anatomic landmarks (Cook et al., 1992).

In brief, the posterior limit was the slice in which the greatest length of fornix was visible whereas the medial limit was the open end of the hippocampal fissure in the posterior/middle portions and the uncal fissure anteriorly. The white matter of the temporal stem and/or cerebrospinal fluid in the temporal horn provided the lateral limit. The head of the hippocampus was distinguished from the overlying amygdala by the presence of the alveus or uncal recess. The alveus, fimbria, and choroid plexus were all included in the measurement, as exclusion would have been too difficult.

The right hippocampus was measured first followed by the left. Hippocampal volumes were derived by multiplying the sum of the cross-sectional area within the traced contour on each slice by the slice thickness and doubled for 3T data (only alternate slices were measured). Manual segmentation of 3T scans took 15 min per hippocampus.

### Automated segmentation

Manual segmentation contours were converted to voxel-based representations, where each voxel was included if the contour enclosed at least half of it. For 3T data, a double slick thickness was used to account for alternate slice segmentation. A template database of high quality 3T scans and bilateral manual segmentations was created by a neurologist (GW) and radiographer (EW) reviewing the scans sequentially until 400 scans without artifacts (e.g., motion) and with reliable manual segmentations had been selected. This is significantly larger than previous template databases used in Alzheimer's disease, but was done in order to encompass a wide range of hippocampal pathologies.

Segmentation was performed using the STEPS (Similarity and Truth Estimation for Propagated Segmentations) algorithm (Cardoso et al., 2013). This is a multi-atlas–based segmentation propagation method based on STAPLE (simultaneous truth and performance level estimation) (Warfield et al., 2004) but adapted to use a local ranking strategy (according to image similarity) for template selection, thus enabling reliable segmentation of hippocampi with variable morphologies, as may occur in HS.

First, group wise templates of the anatomic scans and hippocampal segmentations were created using a previously described method (Rohlfing et al., 2004). The first iteration selects one arbitrary individual image as a reference and registers each of the remaining images to the reference using an affine transformation. Using these transformations, an average image is computed. In the second iteration, all individuals including the initial reference are registered to the average image by nonrigid transformations. A new average image is generated using the new transformations and used as the reference for the following registration iteration. The procedure is repeated until convergence.

Next, the subject scan to be segmented was nonlinearly registered to the group template using Fast Free Form Deformation, and the hippocampal segmentation templates were each propagated to the subject scan to give an approximate location for the hippocampus. A region-of-interest was extracted from the subject scan encompassing this region.

Each scan in the template database was coarsely registered to the region-of-interest, and the most representative 75 subjects were selected on the basis of the highest normalized cross-correlation between the subject scan and the registered template. A finer more accurate registration of these 75 subjects to the region-of-interest was performed, and the same transformations were applied to the manual hippocampal segmentations for these scans.

**Figure 1:**
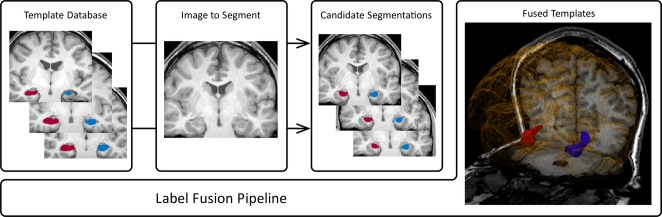
Automated hippocampal segmentation algorithm. The templates that are the most similar to the new image are selected from the template database and fused to produce the new segmentation.

The 15 most similar subjects at each spatial location were selected according to the locally normalized cross-correlation over a Gaussian kernel with standard deviation of 2 voxels and fused using a probabilistic framework that iteratively estimates the most likely true segmentation and performance parameters (Fig. 1). Spatial smoothness was enforced using a Markov Random Field (beta = 0.5).

The entire process, from retrieving the images from the scanner database to the final hippocampal segmentations and volumes of each side, took approximately 30 min per subject using a Linux workstation with a quad-core Intel Xeon processor (Dell, Round Rock, TX, U.S.A.) running at 3·20 GHz with less than a minute of operator time.

### Degree of overlap

The overlap between manual and automated segmentations was quantified using the Dice coefficient, which ranges from 0 (no overlap) to 1 (complete overlap).


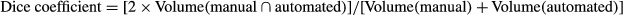


Because manual segmentations were performed on alternate slices but automated segmentations describe each slice, the manual segmentations were resampled to the resolution of the automated segmentations to compute these measures.

Dice coefficients were calculated for the manual segmentations of the 18 healthy controls to assess interrater and intrarater reliability.

### Hippocampal volumes

The automated and manual volumes for the hippocampi on each side were compared by linear regression in PASW Statistics v18 (SPSS; Chicago, IL, U.S.A.), and Bland-Altman plots were used to assess the equivalence of these measurements without correction for intracranial volume.

Clinical practice requires correction for intracranial volume, as hippocampal volumes vary with brain size (Free et al., 1995). Intracranial volume was estimated by applying the same algorithm with a template database of brain segmentations. Linear regression was used to determine the relationship between hippocampal volumes and intracranial volume in the 100 hippocampi of 50 healthy controls. Hippocampal volumes were corrected by the following formula where Grad is the gradient of the regression line and ICV is intracranial volume:


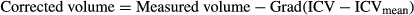


### Volume classification

A scatter plot of sum of corrected automated volumes versus difference in corrected automated volumes (left–right) was used to illustrate the discriminating power of the volumes between different groups, but all other data are presented with uncorrected volumes. The discriminating power was assessed between different clinical classifications (normal, left hippocampal atrophy, right hippocampal atrophy, bilateral hippocampal atrophy) as determined by a neuroradiologist based on visual review, manual hippocampal volumes, and T2 measurements, and secondly between different pathologic classifications (normal hippocampus, left HS, right HS) in those patients who had undergone temporal lobe resection.

## RESULTS

### Template database

The 400 patients (199 male) in the template database had a median age of 34 years (interquartile range [IQR] 26–42), median age of onset of epilepsy 12 years (IQR 7–21), and median duration of epilepsy 18 years (IQR 10–28).

### Degree of overlap (Dice coefficients)

The Dice coefficients for both 3T and 1.5T scans were comparable to the interrater reproducibility in healthy subjects regardless of the size of the hippocampi (Table 1). Linear regression demonstrated that for each 1 cm^3^ reduction in hippocampal volume, the Dice coefficient for 3T scans fell by only 0.028 (left) or 0.030 (right). This minor drop in segmentation performance is expected, as the Dice coefficient is naturally lower for smaller structures. Thus automated segmentation remained accurate in small sclerotic hippocampi.

**Table 1:** Overlap between manual and automated hippocampal segmentation

	Number	Dice coefficients–mean (SD)		
		Left	Right	Both
3T patient scans				
All scans	876	0.844 (0.043)	0.850 (0.036)	0.847 (0.040)
Subset in template database	400	0.854 (0.032)	0.861 (0.031)	0.857 (0.032)
Subset not in template database	476	0.836 (0.049)	0.840 (0.037)	0.838 (0.043)
1.5T patient scans				
All scans	202	0.824 (0.036)	0.830 (0.036)	0.827 (0.036)
3T control scans				
Interrater	18	0.824 (0.023)	0.840 (0.018)	0.832 (0.022)
Intrarater	18	0.893 (0.022)	0.890 (0.025)	0.891 (0.024)

### Hippocampal volumes

**Figure 2:**
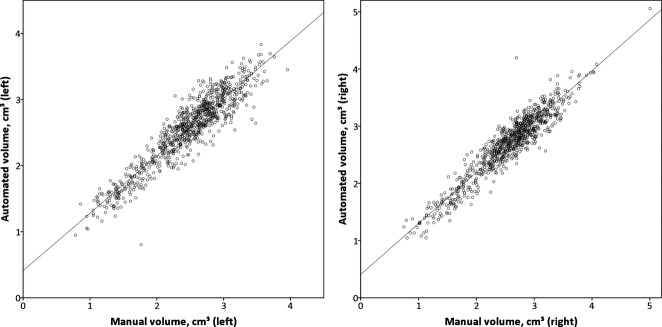
Manual and automated hippocampal volumes on 3T scans. There is good agreement between the volumes for both the left and right hippocampi.

**Figure 3:**
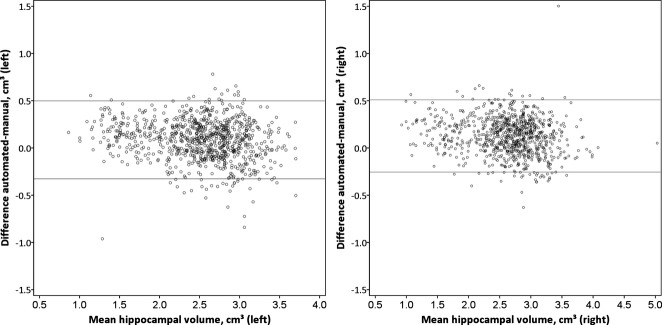
Bland-Altman plots for manual and automated hippocampal volumes on 3T scans. There is no evidence of bias between the methods for both the left and right hippocampi.

At 3T, there were strong correlations between manual and automated volumes for both the left (Pearson correlation coefficient 0.929, one-tailed p < 0.001) and right (0.941, p < 0.001) hippocampi (Fig. 2). The outlier for the left hippocampus reflected inaccurate automated segmentation owing to poor image registration in a subject with an extensive childhood stroke resulting in destruction of the majority of the left hemisphere. On the right, one subject had a particularly large dysplastic hippocampus, whereas the single outlier was a patient with subependymal heterotopia in whom some of the heterotopic gray matter was misclassified as being part of the hippocampus. Bland-Altman plots confirmed no bias between manual and automated measurements (Fig. 3). Similar results were obtained with the 1.5T scans using the 3T template database with correlations of 0.918 (p < 0.001, left) and 0.924 (p < 0.001, right) (Fig. S1).

### Hippocampal volumes and intracranial volumes

Larger hippocampal volumes in healthy controls were associated with larger intracranial volumes, leading to the following equation to correct for the intracranial volume:


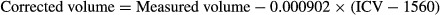


The reference range for hippocampal volumes in these healthy controls based on the mean ± 1.96 standard deviations (SD) was 2.42–3.28 cm^3^.

**Figure 4:**
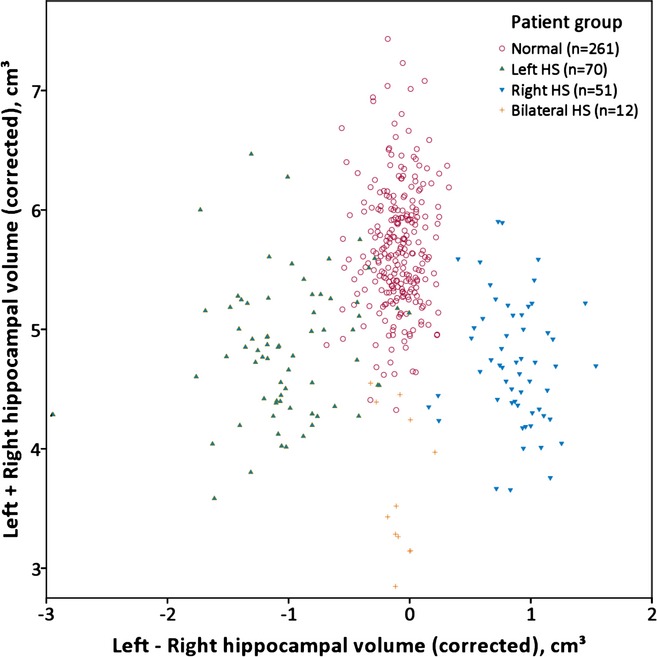
Corrected volumes and asymmetry for subjects in the template database. Scatterplot of sum of left and right hippocampal volumes versus difference between these volumes (corrected for intracranial volume) divided into groups by the prior clinical classification.

The corrected hippocampal volumes in patients are shown split by clinical classification in Fig. 4, with the data summarized in Table 2. Of the 146 subjects who underwent temporal lobe resection involving the hippocampus, those with classical hippocampal sclerosis on histology (n = 95) had hippocampal volumes of 1.12–3.22 cm^3^ (mean 1.90, SD 0.391) and those with normal hippocampi (n = 37) had hippocampal volumes of 2.20–3.39 cm^3^ (mean 2.87, SD 0.255). Fourteen subjects with only end folium gliosis or sclerosis that may not be associated with volume change were excluded. Note, however, that in clinical practice, decisions regarding epilepsy surgery involve many other data as well as hippocampal volumes.

**Table 2:** Corrected hippocampal volumes by group

Group	Left hippocampal volume (cm^3^)	Right hippocampal volume (cm^3^)
For correction of volumes		
Healthy controls (n = 50)	2.84 (0.224)	2.85 (0.216)
By clinical classification		
Normal (n = 261)	2.79 (0.278)	2.89 (0.267)
Left hippocampal atrophy (n = 70)	1.91 (0.395)	2.92 (0.336)
Right hippocampal atrophy (n = 57)	2.79 (0.279)	1.92 (0.309)
Bilateral hippocampal atrophy (n = 12)	1.80 (0.290)	1.89 (0.324)
By operative histology		
Normal (n = 37)	2.80 (0.215)	2.91 (0.254)
Left hippocampal sclerosis (n = 50)	1.90 (0.383)	2.96 (0.287)
Right hippocampal sclerosis (n = 45)	2.80 (0.278)	1.90 (0.404)

## DISCUSSION

### Summary of main findings

We demonstrate robust, automated hippocampal segmentation and volumetry in an epilepsy population, giving reliable identification of hippocampal atrophy in patients with HS. This is crucial for clinical management of epilepsy, particularly if surgical treatment is being contemplated. In addition to determining the presence of atrophy in a potentially epileptogenic hippocampus, the integrity of the contralateral hippocampus can be determined.

A particular feature was a template library of 400 adult patients with epilepsy including the range of severity of HS, from unilateral and focal to bilateral and diffuse. This was created using 3T data and also gave excellent results on data acquired on a different 1.5T scanner. This suggests that the algorithm should remain robust across different platforms with different hardware and pulse sequences.

### Online automated segmentation

We provide for the first time a free online web-based automated hippocampal segmentation algorithm based on this large template database. Previous databases for hippocampal segmentations have been typically small and include only healthy individuals (Internet Brain Segmentation Repository, 18 healthy subjects, http://www.cma.mgh.harvard.edu/ibsr/; LONI Probabilistic Brain Atlas, 40 healthy subjects, http://www.loni.usc.edu/atlases/LPBA40; Hammers atlas, 20 healthy subjects, http://www.brain-development.org/). The Alzheimer's Disease Neuroimaging Initiative (ADNI, http://www.adni-info.org) provides a larger dataset, but segmentations are semi-automated rather than entirely manual. For epilepsy, there is a single database of 40 patients with epilepsy and 10 healthy controls acquired on two different scanners with labels provided for half of the scans (Jafari-Khouzani et al., 2011).

### Previous algorithms for automated hippocampal segmentation in epilepsy

Several groups have applied automated segmentations in patients with epilepsy. A common theme is inferior performance of automated segmentation in diseased hippocampi. A single template used in five patients with mesial TLE achieved a Dice coefficient of 0.83 in healthy hippocampi but only 0.67 on the sclerotic side (Hogan et al., 2000). A multi-atlas technique in nine patients with mesial TLE achieved a Dice coefficient of 0.83 on the healthy side but 0.76 in the diseased hippocampi (Hammers et al., 2007). A recent entropy-based segmentation algorithm using multiple atlases achieved a Dice coefficient of only 0.72 in 46 subjects with mesial TLE. The atlases for all these studies were derived from healthy subjects.

A comparison of the automated methods of FreeSurfer (single atlas) and FMRIB Software Library (FSL) (shape and appearance-based model) gave Dice coefficient of 0.71–0.73 in controls, but 0.62–0.66 in mesial TLE (Pardoe et al., 2009). The best results to date have come from a semi-automated method implemented in the ANTS toolkit that achieved a Dice coefficient of 0.83 in diseased hippocampi (Pluta et al., 2009), and an automated method with hybrid constraints that achieved a Dice coefficient of 0.84 in a mixed group (8 controls, 15 with epilepsy including 8 HS) (Chupin et al., 2009).

### Performance of the present algorithm

In individuals with hippocampal pathology, template selection is critical (Avants et al., 2010). Our technique uses a large template database to encompass a variety of hippocampal pathologies. This is critical when around 40% of patients with TLE have an atypical shape or position of the hippocampus (Bernasconi et al., 2005). The templates for the most similar scans within this database are used to produce a segmentation of a new scan, thus allowing accuracy to be maintained regardless of pathology.

The Dice coefficient of the automated method equals (1.5T, 0.827) or exceeds (3T, 0.844) the interrater Dice coefficient (0.832). Therefore the automated segmentation introduces no more variability than that seen between different raters when applied to scans from two different scanners and field strengths. Unlike previous methods, the Dice coefficient does not markedly decline with small sclerotic hippocampi. The method was robust with only three poor results among over 1,000 scans at different field strengths. However, it remains important to review any automated results obtained to ensure that they are reasonable. The automated protocol has the further great advantage that the possibilities of human error and inconsistency are avoided.

### Limitations

Assessment of the Dice coefficient requires segmentations in the same format, so it was necessary to convert the manual contour-based representations to voxel-based segmentations first. These differences may explain the slight offset of the regression lines between manual and automated volumes and demonstrate that it is necessary to produce new reference ranges for the automated volumes, with correction for intracranial volume.

Manual segmentations of 3T scans were performed on alternate slices, which may reduce the maximum Dice coefficient that can be achieved. Although this limits the resolution of the template database, this effect should be mitigated by the merging of multiple segmentations to produce a new automated segmentation. The technique presented relies solely on volume-based analysis, but some recent approaches suggest there may be merit in including shape-based analysis (Coupe et al., 2012).

### Future application

We have made the algorithm available freely online so that individual T_1_-weighted MRI scans may be subject to automated hippocampal volumetry for the diagnosis of unilateral and bilateral hippocampal atrophy in those with epilepsy. This will result in reliable hippocampal volumetry being available globally, with consequent greatly improved evaluation of those with epilepsy. Finally, with an appropriate template library, our hippocampal volumetry technique can be extended to other populations, such as patients with dementia or a pediatric population.

## SUPPORTING INFORMATION

Figure S1 Manual and automated hippocampal volumes on 1.5T scans.
