# Supplementary figures and images for: Automated hippocampal segmentation in patients with epilepsy: Available free online
Source: Epilepsia. 2013 Oct 23;54(12):2166–73. doi: 10.1111/epi.12408 (PMC3995014; doi:10.1111/epi.12408)

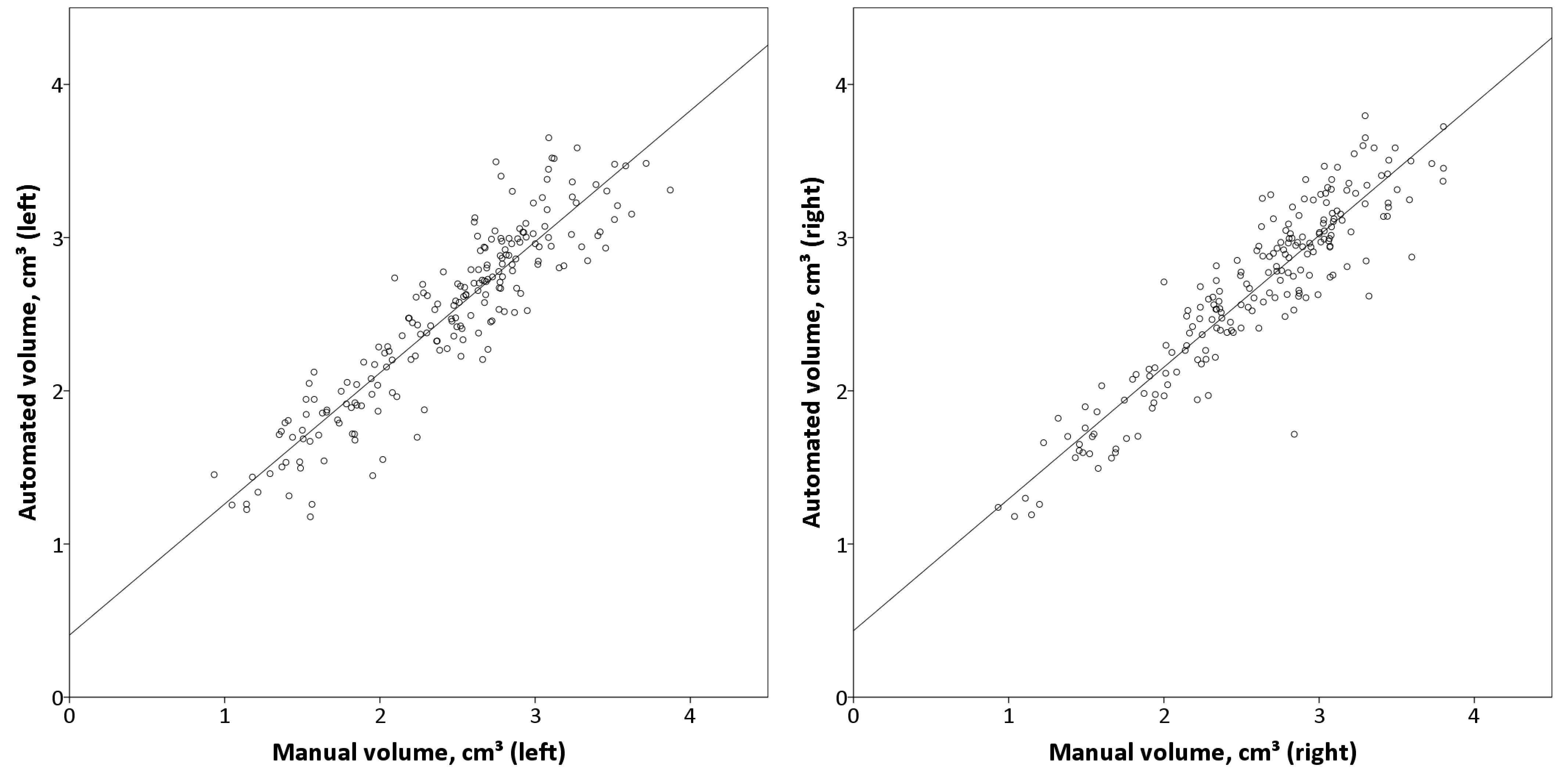

Supplement: Figure S1 — Manual and automated hippocampal volumes on 1.5T scans. [file epi0054-2166-sd1.tif]
