# Supplementary material for: Automated hippocampal segmentation in patients with epilepsy: Available free online
Source: Epilepsia. 2013 Oct 23;54(12):2166–73. doi: 10.1111/epi.12408 (PMC3995014; doi:10.1111/epi.12408)
Supplement: Supplementary file 2 [file epi0054-2166-sd2.pptx]

## Slide 1
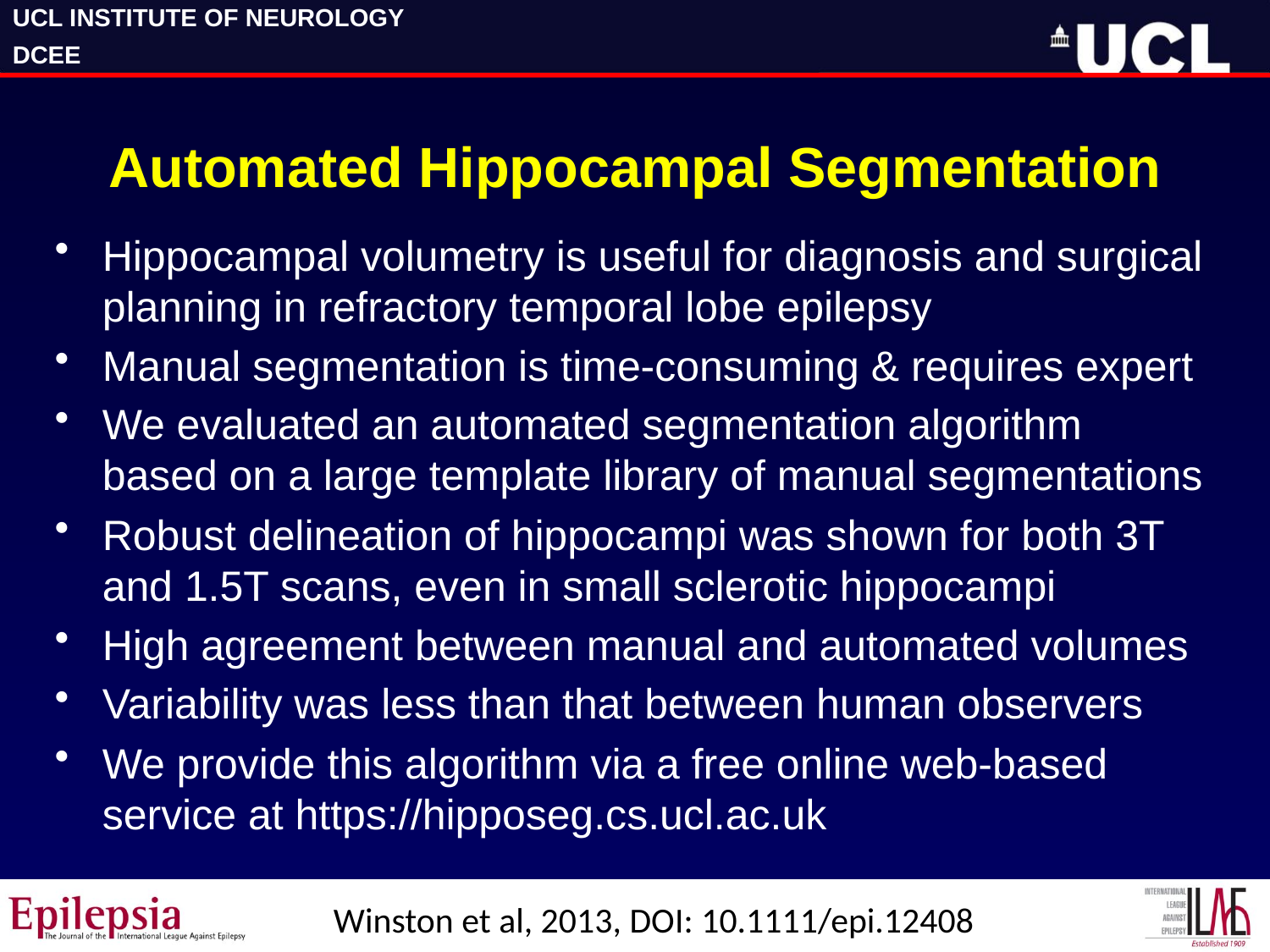

# Automated Hippocampal Segmentation
Hippocampal volumetry is useful for diagnosis and surgical planning in refractory temporal lobe epilepsy
Manual segmentation is time-consuming & requires expert
We evaluated an automated segmentation algorithm based on a large template library of manual segmentations
Robust delineation of hippocampi was shown for both 3T and 1.5T scans, even in small sclerotic hippocampi
High agreement between manual and automated volumes
Variability was less than that between human observers
We provide this algorithm via a free online web-based service at https://hipposeg.cs.ucl.ac.uk
Winston et al, 2013, DOI: 10.1111/epi.12408
